# Supplementary material for: Structural Optimization of Vertically-Stacked White LEDs with a Yellow Phosphor Plate and a Red Quantum-Dot Film
Source: Nanomaterials (Basel). 2022 Aug 18;12(16):2846. doi: 10.3390/nano12162846 (PMC9414739; doi:10.3390/nano12162846)
Supplement: Supplementary file 1 [file nanomaterials-12-02846-s001.zip › nanomaterials-1872416-supplementary.pdf]

nanomaterials

MDPI

Supplementary Materials

Structural Optimization of Vertically-Stacked White LEDs with a Yellow Phosphor Plate and a Red Quantum-Dot Film

Seung Chan Hong and Jae-Hyeon Ko *

School of Nano Convergence Technology, Nano Convergence Technology Center, Hallym University, Chuncheon 24252, Gangwondo, Korea

* Correspondence: hwangko@hallym.ac.kr.

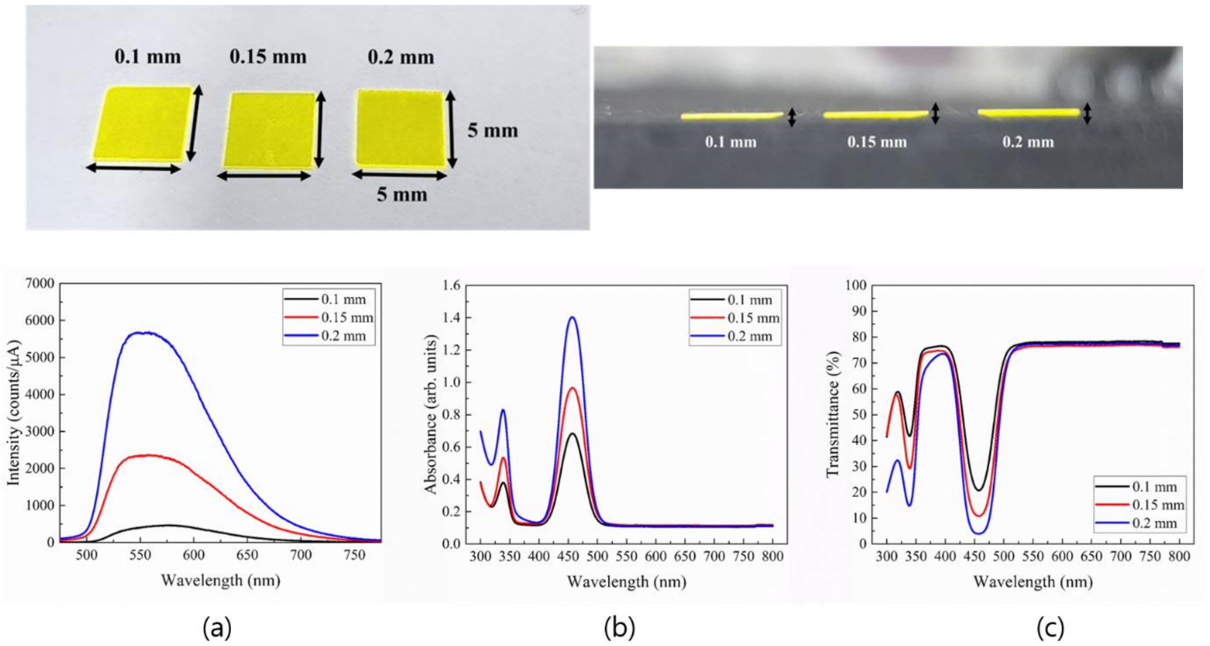

Figure S1. Photos and optical characteristics of YAG yellow phosphor plates: (**a**) PL spectra, (**b**) absorbance and (**c**) transmittance spectra at several thicknesses.

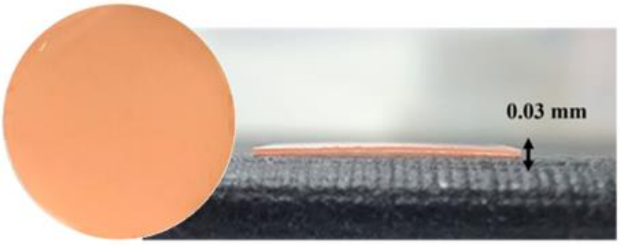

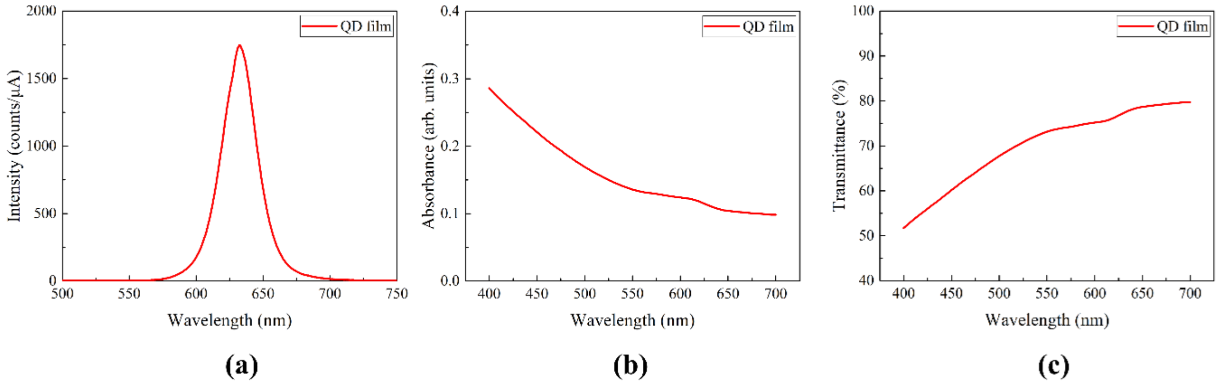

Figure S2. Photos and optical characteristics of QD films: (**a**) PL spectra, (**b**) absorbance and (**c**) transmittance spectra.

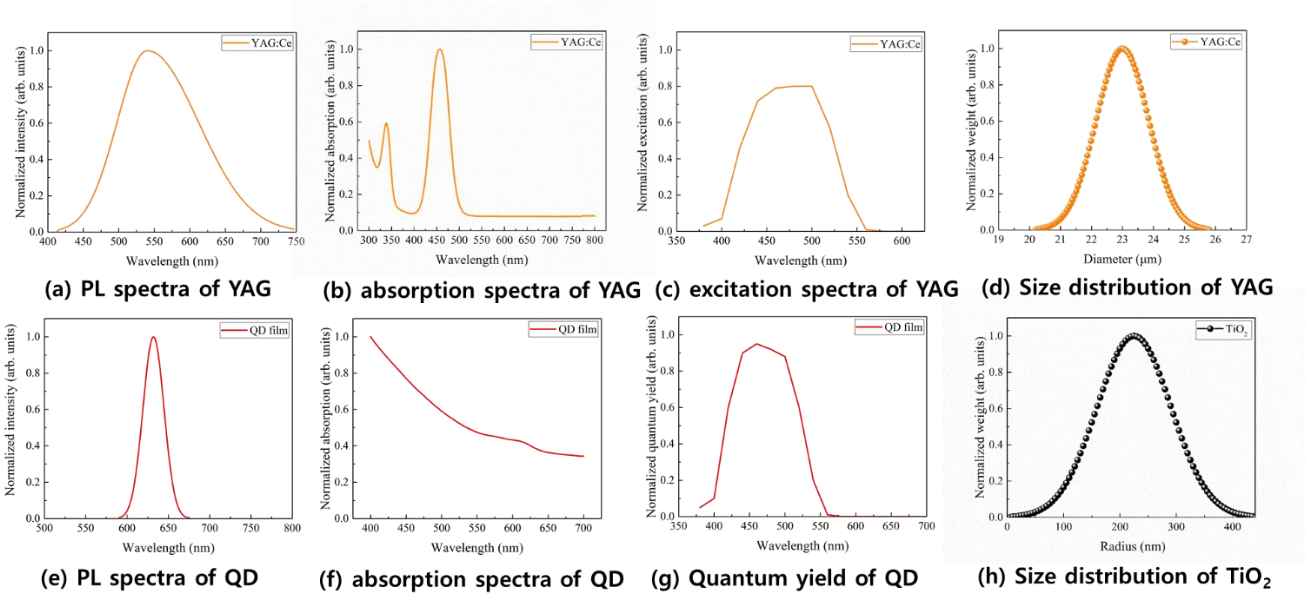

Figure S3. (**a**–**h**) PL, absorption, excitation spectra and quantum yield in addition to the size distributions of phosphors and TiO_2 particles used in the simulation.

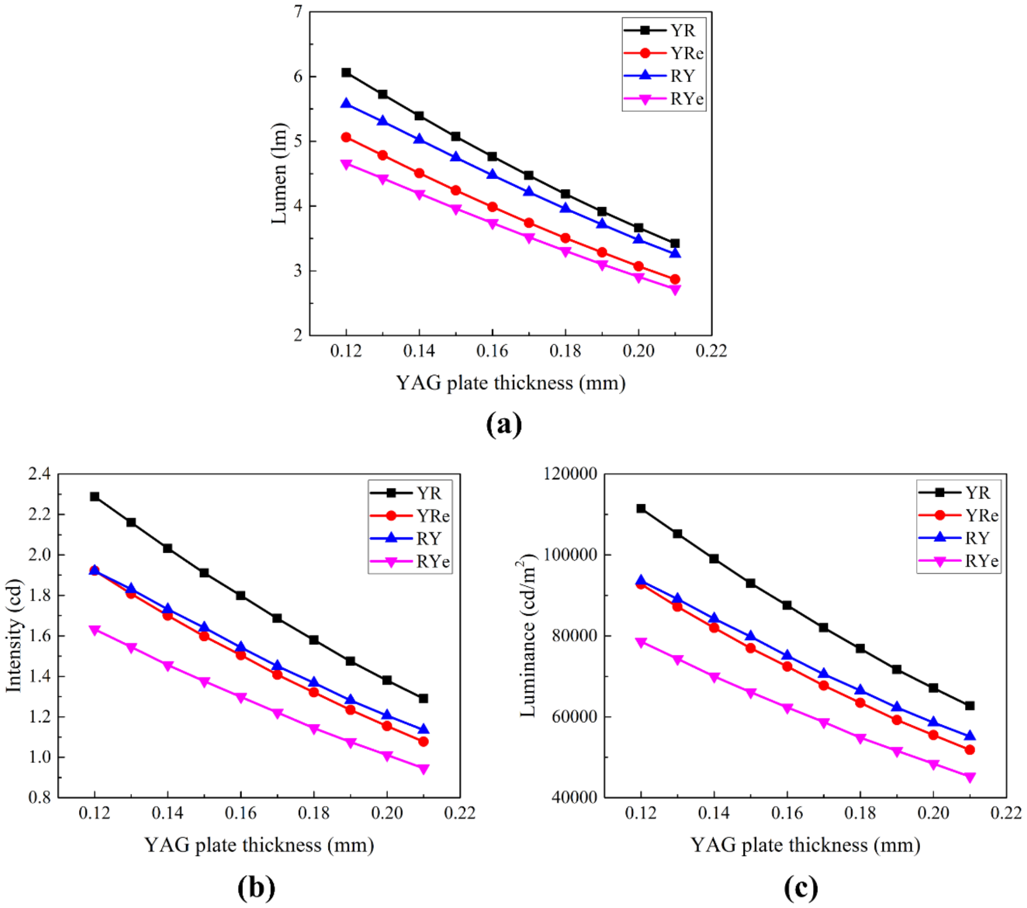

Figure S4. The thickness dependence of (**a**) the luminous flux, (**b**) the luminous intensity, and (**c**) the luminance of the white LEDs with four configurations.
